# Irreversible Electroporation in Pancreatic Cancer—An Evolving Experimental and Clinical Method

**DOI:** 10.3390/ijms24054381

**Published:** 2023-02-23

**Authors:** Agnieszka Gajewska-Naryniecka, Urszula Szwedowicz, Zofia Łapińska, Julia Rudno-Rudzińska, Wojciech Kielan, Julita Kulbacka

**Affiliations:** 1 Department of Molecular and Cellular Biology, Faculty of Pharmacy, Wroclaw Medical University, Borowska 211A, 50-556 Wroclaw, Poland; agnieszka.gajewska-naryniecka@umw.edu.pl (A.G.-N.); urszula.szwedowicz@student.umw.edu.pl (U.S.); zofia.lapinska@student.umw.edu.pl (Z.Ł.); 2 2nd Department of General Surgery and Surgical Oncology, Wroclaw Medical University, Borowska 213, 50-556 Wroclaw, Poland; julia.rudno-rudzinska@umw.edu.pl (J.R.-R.); wojciech.kielan@umw.edu.pl (W.K.); 3 Department of Immunology, State Research Institute Centre for Innovative Medicine, Santariškių 5, 08410 Vilnius, Lithuania

**Keywords:** pancreatic cancer, irreversible electroporation, immune response

## Abstract

Pancreatic cancer has no symptoms until the disease has advanced and is aggressive cancer with early metastasis. Up to now, the only curative treatment is surgical resection, which is possible in the early stages of the disease. Irreversible electroporation treatment offers new hope for patients with unresectable tumors. Irreversible electroporation (IRE) is a type of ablation therapy that has been explored as a potential treatment for pancreatic cancer. Ablation therapies involve the use of energy to destroy or damage cancer cells. IRE involves using high-voltage, low-energy electrical pulses to create resealing in the cell membrane, causing the cell to die. This review summarizes experiential and clinical findings in terms of the IRE applications. As was described, IRE can be a non-pharmacological approach (electroporation) or combined with anticancer drugs or standard treatment methods. The efficacy of irreversible electroporation (IRE) in eliminating pancreatic cancer cells has been demonstrated through both in vitro and in vivo studies, and it has been shown to induce an immune response. Nevertheless, further investigation is required to assess its effectiveness in human subjects and to comprehensively understand IRE’s potential as a treatment option for pancreatic cancer.

## 1. Pancreatic Cancer Classification

Pancreatic cancer (PC) is one of the deadliest cancers, the treatment of which is still a challenge for scientists. Despite little improvement in the past two decades, the prognosis for PC remains still poor with a 5-year survival rate of only 8%. Due to the lack of clear early symptoms, c.a. 30% of patients are diagnosed with locally advanced pancreatic carcinoma (LAPC) and 50% with metastatic pancreatic cancer (mPC) [1]. Therefore, PC has a relatively low incidence and high mortality. A surgical resection followed by adjuvant treatment is the only chance to cure PC. However, this is only possible in about 10% of cases, and some patients, after resection, develop metastases within a few years of surgery [1,2]. Pancreatic cancer (PC) is classified into several different types based on the type of cells involved and the characteristics of cancer. The most common types of pancreatic cancer are [3,4]:

- Adenocarcinoma is the most common type of pancreatic cancer, accounting for about 95% of cases. It arises from glandular cells in the pancreas that produce digestive enzymes.
- Neuroendocrine tumors: these tumors arise from cells in the pancreas that produce hormones and are responsible for regulating various bodily functions. Neuroendocrine tumors can be benign (non-cancerous) or malignant (cancerous).
- Sarcomas are rare tumors arising from connective tissue cells in the pancreas.
- Acinar cell carcinomas arise from cells in the pancreas that produce digestive enzymes.

Pancreatic cancer is generally classified as stages I to IV, based on the extent of the cancer and whether it has spread to other parts of the body. Stage I cancer is the least advanced, and stage IV is the most advanced. Independently on the type of this cancer, the most critical factors are the size and location of the tumor, the presence of cancer cells in nearby lymph nodes, and the extent to which cancer has spread to other parts of the body [3,4]. Thus, the treatment of this cancer is still challenging, and new methods or improvements of currently used protocols are required. In response come physical methods, including those based on pulse electric fields (PEFs).

## 2. Principals of Irreversible Electroporation

Electroporation is the phenomenon that relies on the formation of pores in cell membranes upon application of a high-amplitude electric field of sufficient duration. Depending on the pulsed electric field (PEF) parameters, the process can be reversible when membrane integrity is quickly recovered or irreversible, leading to permanent changes in the cell because of severe damage to the cell membrane structure. Irreversible electroporation (IRE) is a type of ablation therapy used to treat pancreatic and other types of cancer. It involves the use of high-voltage, low-energy electrical pulses to create permanent unsealing of the cell membrane, causing damage and, finally, cell death [5,6,7].

Over the last decades, many studies have focused on analyzing the effectiveness of irreversible electroporation (IRE) as a potential new treatment strategy for different carcinomas. The ablation area created by applied IRE is sharply delineated, allowing for strict control of its boundaries [8]. Moreover, IRE is characterized by a non-thermal mechanism, no collateral heat injuries to surrounding tissues, and no “heat-sink” effect on blood vessels near the ablation area [9,10]. As was mentioned by Rubinsky et al., 2007 [11], IRE is an ablation modality, which can effectively permeabilize cell membranes and induce necrosis. IRE creates irreversible nanoscale defects in the cell membrane when exposed to a pulsed electrical field with appropriate parameters, resulting in the loss of homeostatic mechanisms in cells. Unlike other ablation techniques, scientists indicated that IRE does not destroy collagenous and other protein or lipid-based structures [11,12,13,14].

These features allow damage to determine significant vital structures encasements, such as blood vessels or nerves, that would otherwise survive other thermal ablation methods.

## 3. IRE on Pancreatic In Vitro Models

Davalos et al., 2005 [15] demonstrated for the first time that irreversible electroporation, in addition to classical surgery and chemotherapy, could be a helpful tool for better results in cancer therapy. Irreversible electroporation (IRE) is a technique that uses electrical pulses of high voltage and amplitude, which lead to lethal cell damage [16]. Previously ignored, it is becoming a superior procedure for unresectable tumors located amid delicate, well-innervated, and blood supply structures such as the pancreatic parenchyma with portal vein, visceral artery, and bile ducts [17]. IRE effectiveness does not depend on the emission of energy in the form of heat; thus it has an advantage over other ablative techniques, especially in the case of organs rich in blood vessels, such as the pancreas, where circulating blood causes an unfavorable heat sink effect that reduces the area of destruction of tumor-transformed tissue [9]. They have also been proven to spare delicate scaffolds and cause negligible damage to normal tissues.

In broad terms, it is conventionally assumed that the electric field’s strength should be higher than 1000 V/cm to achieve irreversible electroporation [18]. However, it is essential to note that different types of cells have various sensitivities to electrical impulses, which requires careful planning of the IRE [19]. In such cases, a tool that allows the in-silico determination of the effects of therapy depending on the electroporation parameters used, based on mathematical modeling called the Peleg–Fermini model, is helpful [20]. It is excellent for the treatment planning of homogeneous structures. However, in the case of structures with variable echogenicity, more data are needed about the organ in question and its microenvironment, which can only be provided by in vitro and in vivo studies. Otherwise, when applying local therapy to organs such as the pancreas, it is possible to perfuse the florid vessels and bile ducts or lead to pancreatitis [21]. 

IRE is currently being successfully used to treat inoperable pancreatic cancer under the commercial name NanoKnife [22]. It is a stand-alone anticancer technique when tumor resection is impossible or for margin accentuation [23]. This method has many advantages, the most important of which are the sparing of surrounding elements, such as the extracellular matrix, and the death of cancer cells by beneficial apoptosis without causing inflammation. Unfortunately, the size of pancreatic cancer or the tumor’s location often makes it impossible to achieve a homogeneous electric field, which leads to reversible electroporation, the incomplete achievement of the desired goal. Therefore, research is currently being conducted to improve IRE, mainly by synergizing it with other well-known methods. The following examples of combination therapy options have one thing in common. Their target is not a solid tumor located in an inoperable site, as this is addressed by IRE alone. Efforts are being made to find complementary elements so that the tumor microenvironment can be optimally impacted and disseminated tumor cells that are out of reach of the electrode can be effectively eradicated.

Studies using anticancer drugs such as gemcitabine and FOLFIRINOX are particularly promising. According to the data presented by Bhutiani et al., 2020 [24], the use of stand-alone IRE results in a two-times decrease in the proliferation of tumor cells of the pancreas lines S2013 and Panc-1, and treatment with anticancer drugs results in a three-times decrease in the test performed compared to the control. The combination of both therapies reduced cell growth and proliferation by at least six times. However, contemporary trends favor the use of IRE with other immunomodulatory methods, which will ultimately be safer for patients, especially in the context of the risk of pancreatitis. Sun et al., 2021 [25], reported a novel combination of immunotherapy and electroporation using oncolytic viruses. This combination has many benefits, as all cancer cells subjected to IRE are infected with the virus and killed via ROS-dependent apoptosis by impacting the PI3K/Akt pathway [25]. Electroporation causes an increased influx of M1 viruses into the target site by increasing the permeability of both the vascular network and the cell membranes of neoplastic cells, as well as local activation of the immune system. In addition, the combined treatment enhanced the cell death of PC cells. M1 virus monotherapy reduced the survival of cancer cells to about 60%, and with the addition of electroporation, the survival rate was only 10% for the AsPC-1 cell line at 1500 V/cm. IRE is also used in anti-PD1 immune checkpoint blockade therapy. The electrical impulses have two key effects. It reduces the immunosuppressives of the tumor microenvironment and increases its immunogenicity by activating danger-associated molecular patterns (DAMPs) and dendritic cells. This method, combined with the supply of PD-1, induces the release of DAMPs and selectively activates CD8+ T lymphocytes [26].

It also appears that manipulation of IRE parameters alone causes the desired and controlled activation of the immune system. It has been shown that the mere application of IRE with a higher frequency of electrical pulse generation triggers cellular and humoral immune responses [27]. This method, called high-frequency irreversible electroporation (H-FIRE), results in greater control of the type of cell death that translates into the induction of an antitumor immune response [28]. Studies conducted on 3D cell culture of the human pancreatic adenocarcinoma line BxPC-3 suggest that appropriate manipulation of parameters alters the dynamics of cell death in favor of those dying a regulated death by reducing the population of cells that die a random death. In the case of classical IRE, this has not yet been achieved. It should also be emphasized that in this experiment, higher-frequency electrical impulses were used, which were also bipolar. It has been proven that they are more beneficial and safer than monopolar ones because they require a 2.5 times lower electric field to induce cell death [29]. H-FIRE is currently being touted as a promising successor to NanoKnife, mainly because it does not cause strong muscle contractions, unlike IRE, which does not require cardiac synchronization and does not increase the risk of ventricular arrhythmia [21]. 

Adapting the appropriate electroporation parameters to the cells is one of the more difficult tasks in therapy planning. The determination should include not only the baseline threshold but also the number of pulses, their frequencies, and their durations. The selection of baseline parameters during therapy planning is a complex process that takes into account various factors, including the type of cells being treated, their physiological and morphological characteristics, and the intended therapeutic outcome. To determine the baseline parameters, researchers and clinicians typically use a combination of in vitro and in vivo experiments, as well as computational modeling and simulations [17,22]. IRE generators (e.g., NanoKnife) are often supported by appropriate software, which optimizes the electric pulse delivery to the targeted tissue. Once the baseline parameters are determined, they can be adjusted based on the specific requirements of each patient, taking into account factors such as age, overall health, and previous medical history. The ultimate goal is to find the optimal parameters that produce the desired therapeutic effect with the minimum amount of electrical energy while avoiding damage to healthy tissues.

Shao et al., 2018 [19] proved that by maintaining the same dose of electrical energy while manipulating the length and frequency of the pulses, different effects could be achieved [19]. They chose 51 pulses of 50 μs duration as the baseline parameters. In vitro studies have shown that a 10-fold reduction in the frequency of electrical pulses results in a reduction in the survival of AsPC-1 cells by approximately 15–25%, depending on the voltage applied. When a 30 s interval was added every 17 pulses, better results were obtained, although these were less significant than with frequency manipulation. Variations in cell survival also occur between other pancreatic cancer cell lines. For the same parameters, the discrepancy in cell survival between the KPC and AsPC-1 lines was as high as ~40% (75% and 35%, respectively). Similar conclusions were reached by Han et al., 2022 [20] who showed the importance of the number of pulses to be no less than the voltage used. Using ten pulses at 2000 V/cm, the cell survival rate of the PANC-1 line was comparable to that of 40 pulses at 1000 V/cm. The same survival rate for the MIA PaCa-2 cell line was observed for cells treated with ten pulses at 1500 V/cm and 40 pulses at 1000 V/cm [20].

A significant influence on IRE’s efficacy for treating pancreatic cancer lesions is the environment in which cells live and proliferate. Therefore, the culture medium is an important variable influencing the effect of electroporation. The use of different media when culturing the same cell line produced different results in subsequent experiments. It has been shown that the more inorganic and vitamin-rich the medium, the greater the resistance of cells to the IRE used [19]. Comparing the effects of PBS, DMEM, and RPMI at 750 V/cm, the effect of electroporation varied the most, with cell survival rates are 50, 74%, and 98%, respectively. At 1000 V/cm and 1250 V/cm, the difference between PBS, DMEM, and RPMI became more pronounced (18, 22, 86%, and 10, 10, and 74%, respectively). These results suggest a high effect of medium supplementation with biotin, vitamin B12, and acid on pancreatic cancer cells and, consequently, on the effect of pulse therapy, which can be partly explained by the different conductivities and osmolarities of the media [30].

Pancreas functions are significantly influenced by diet, especially carbohydrate supply. Population-based studies have confirmed that obesity and diabetes are the two most crucial non-inherited risk factors for pancreatic cancer incidence [31]. Therefore, when planning cancer therapy using electroporation, special attention should be paid to the patient’s glycemic results. In in vitro studies, the effect of glucose concentration on the subsequent survival of cells treated with electrical pulses has been shown in addition to the previously mentioned medium effect related to conductivity and pH [19]. The higher the glucose concentration, the worse the efficiency. There are also significant values classified as normal human glycemia (80–125 mg/dL) and those that indicate diabetes. With results close to these physiological ones, the IRE was about 16%, and for higher values classified as pathological, it was between 17 and 30.5%. 

## 4. IRE in Clinical Practice of Pancreatic Cancer

Irreversible electroporation (IRE) found its clinical application as a novel non-thermal ablation technique for solid tumors. It is suitable for application in the proximity of sensitive structures such as vessels, bile ducts, or intestinal walls. However, IRE was initially considered an undesirable side effect of reversible electroporation and was utterly ignored in cancer therapy. Davalos et al., 2005 [15] proposed the usage of IRE as monotherapy to destroy tissue in a non-thermal manner [15]. When the results of IRE treatment in animal models showed promising outcomes, IRE started to be applied in human patients after 2009 when FDA approved the NanoKnife IRE delivery system. Pech et al., 2011 [32] reported the first in vivo use of IRE for ablating renal cell carcinoma in 2010, showing feasibility and safety in patients with kidney tumors [32]. Since then, numerous preclinical and clinical studies on IRE have been conducted on different cancer types, including kidney [32,33], liver [34,35], prostate [35,36], cholangiocarcinoma [37], and pancreas [38]. Here, we review clinical studies of IRE in pancreatic cancer in the past decade. The first report on the application of the IRE procedure in PC came in 2012 [39] involving a prospective multi-institutional pilot study on 27 locally advanced pancreatic cancer (LAPC) patients. In the study, 26 patients underwent an open procedure of IRE, and one was treated percutaneously. During the 90-day follow-up, they reported only one case of death, and no patient had clinical signs of pancreatitis or fistula formation with successful ablation of pancreas tumors. This pilot study showed the feasibility and safety of IRE ablation of unresectable LAPC, indicating it as one of a few options available to patients with locally advanced pancreatic cancer [39] The results of this study encouraged scientists’ further study of irreversible electroporation ablation of pancreatic cancer, showing the unique benefits of IRE compared to other focal therapies. Table 1 reviews clinical studies of IRE in pancreatic cancer patients.

Although advantages of the IRE procedure, there are some limitations of the IRE procedures when coming into the clinic. Due to the long duration (1–100 ms) of electrical pulses, muscle contractions can occur, and there is a risk of generating cardiac arrhythmias. There is a need for general anesthesia with the administration of neuromuscular blocking agents and the use of an electrocardiogram (ECG) synchronizer to synchronize the delivery of IRE pulses with the refractory period of cardiac rhythm [49]. IRE should not be considered a minimally invasive treatment because severe adverse events can occur. However, a growing number of studies show its effectiveness in local control of disease and better overall survival and improved quality of life of PC patients [45,48].

## 5. What Drugs Are Used with IRE?

Due to the poor response of pancreatic tumors to chemotherapy, resulting from poor vascularization of the tumor and often its resistance to cytostatic drugs, systemic treatment based on chemotherapy leads to unsatisfactory results, which usually do not significantly affect the length and quality of life of patients. Gemcitabine is the drug of choice for metastatic or recurrent PC. Several chemotherapeutic agents have been added to this regimen, including capecitabine, erlotinib, and nanoparticle albumin-bound paclitaxel (nab-paclitaxel). The phase III study also showed improved survival with the Folifirinox regimen (5-fluorouracil, leucovorin, irinotecan, and oxaliplatin) in metastatic PC compared to gemcitabine alone [2,50,51]. Unfortunately, the latest treatments still exhibit considerable toxicity and adverse effects. An alternative, in this case, seems to be electrochemotherapy in combination with standard chemotherapy or calcium ions, which in recent years has developed in treating unresectable tumors or those that do not respond to standard therapeutic methods.

**Figure 1:**
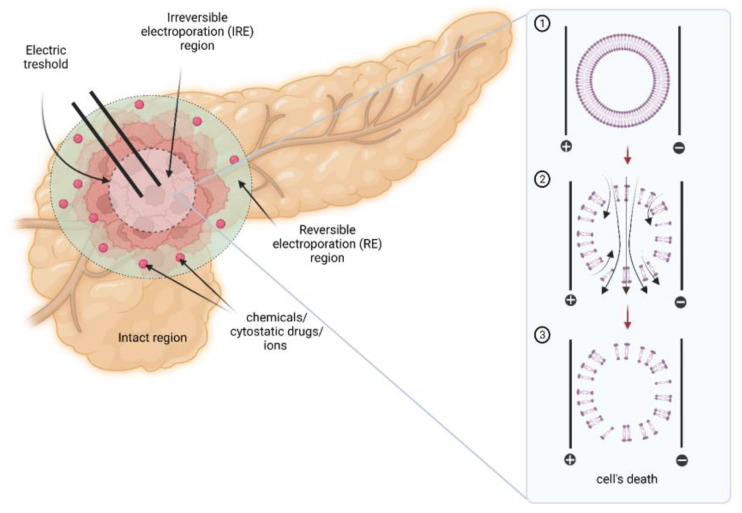
The representation of the IRE activity and action when used in a pancreatic tumor demonstrating tissues’ regions of IRE and RE. (1) lipid cell membrane before IRE exposure; (2) lipid cell membrane during IRE exposure; (3) cell membrane destruction and cell death post-IRE [9,52].

Despite its promising results, scientists point out one critical disadvantage of IRE as a potential cancer treatment method, namely, its non-effectiveness in ablation of the entire area of larger (i.e., >3 cm diameter) tumors [9]. This limited effectiveness may result from the fact that IRE alone induces temporary electroporation of the ablated tissue localized further away from electrodes (Figure 1) [52]. The magnitude of the electric pulse gradually decreases as the distance between the cells and the electrode increases.

Considering such a challenge, a growing number of studies investigate the effectiveness of IRE support by adding chemical substances or drugs. The theory assumes that the delivery of such molecules to transiently electroporated areas will allow the induction of cell death there, consequently increasing the effectiveness of IRE-based therapy. The studies noted in the available literature involve the use of conventional cytostatic drugs, including cisplatin, fluorouracil, and calcium ions.

The phenomenon of electrochemotherapy (ECT) was first described by Mir et al. in 1991 [53]. ECT constitutes the exposure of cell membranes to high-intensity, well-defined electric pulses preceded by intravenous or intratumoral drug administration [54]. Carrying out electroporation (EP) while administering chemotherapy drugs enhances their cytotoxicity and reduces the amount needed to achieve the same or improved therapeutic effect against cancer cells. This advantage is significant regarding the number of reported side effects and the quality of patients’ life. Until now, electroporation (EP) protocols involved in ECT predominantly included parameters defined as reversible electroporation (RE), leading to temporary cell membrane permeabilization. It should be noted that the extent of EP mostly depends on the number of pulses and their duration time. Therefore, IRE using higher numbers and magnitudes of applied electrical pulses is more comprehensive.

Moreover, research over the last two decades allows for defining the main challenges associated with EP-based therapies. Those modalities prevent metastases after treatment and painful muscle contractions during the procedure [55,56]. Therefore, the treatment must be performed using general anesthesia [57], simultaneous observation of electrocardiogram [58,59], and prior administration of muscle relaxants. The replacement of RE by irreversible protocols may shorten the duration of the pulses’ time and, therefore, more selective therapy. Moreover, IRE alone may trigger a significant immunomodulatory effect [60].

Neal et al., 2014 [60] observed an enhanced (2–3 times) cytotoxic effect of IRE combined with ECT chemotherapeutics (carboplatin and bleomycin) in canine J3T glioma cells and human U-87 malignant glioma (MG) compared to IRE alone [61]. The authors indicated that apoptosis plays an important role in the effectiveness of combinatorial treatment.

The available literature reports that adding IRE protocols to cytotoxic agents may help to deal with the growing problem of resistance to chemotherapy. Saczko et al., 2014 [61] analyzed the influence of electroporation (1–3 kV/cm ’5 pulses 50 μs duration, frequency 1 Hz) on human ovarian clear-cell carcinoma cell line (OvBH-1) and epithelial ovarian carcinoma cell line (SKOV-3), both cisplatin-resistance [62]. The applied treatment showed promising effects on both ovarian cell lines. Moreover, the recovery of normal cells has been noted 72 h after therapy. It was also demonstrated that IRE improves the passage of gemcitabine (GEM) into tumor cells within the RE area, inducing tumor cells’ death beyond the region possible with IRE alone in vitro and in vivo [63]. Scientists also observed an enhanced drug delivery effect with FOLFIRINOX combined with IRE compared to GEM, GEM + IRE, and FOLFIRINOX (5-fluorouracil 1.8 mg/L; leucovorin 1.7 mg/L; irinotecan 0.315 mg/L; oxaliplatin 0.425 mg/L) alone [24]. Moreover, the human subject portion of this work is conducted as a clinical trial (#NCT03484299; Table 2). The available clinical trials utilizing the IRE method are listed in Table 2. Ma et al., 2020 [63] pointed out that the heterogeneity of malignant tumors challenges the penetration of unhealthy tissue by conventional chemotherapy agents [64]. Therefore, the authors decided to elucidate whether IRE may be used as an adjuvant to chemotherapy (GEM; 1 mg/m^2^). IRE successfully enhanced the cytotoxic effect of chemotherapy (CT) for patients with locally advanced pancreatic cancer (LAPC). IRE + GEM therapy turned out to be safe and effective, with fewer complications than standard CT using GEM.

Another idea analyzed intensively by researchers is the support of IRE therapy by adjuvant administration of supraphysiological concentration of calcium ions (Ca^2+^) [52]. As mentioned above, alone IRE treatment divides the ablation area into three sectors: cells exposed to irreversible permeabilization and those that may survive therapy. The proposed combinatory could cause the cells’ death within the IRE area due to the loss of homeostasis triggered by the efflux of cells’ components through the permanent electroporated membrane. Calcium ions enable accentuation of the treatment area without applying additional energy by distributing the homeostasis of cells localized within the area of reversible electroporation (Figure 1). The second mechanism of damage is known as calcium electroporation (CaEP). The massive influx of Ca^2+^ results in homeostasis disturbances. In order to regain balance, the cell activates the ATP-dependent exchangers and pumps to remove excess ions outside the cell [65,66]. Additionally, the electrochemical gradient essential for ATP synthesis is disturbed due to calcium overload, and consequently, ATP reservoirs are depleted, triggering cell death. Studies conducted so far indicated that CaEP is safer and more effective than conventional chemotherapy and even ECT [67,68,69,70,71].

Wasson et al., 2017 [52] examined the effectiveness of IRE combined with calcium chloride (CaCl_2_) solutions (1 and 5 mM) on glioblastoma cells in 3D collagen scaffolds [52]. In this study, the delivered IRE consisted of 80 pulses, 450 V/cm, a frequency of 1 Hz, and a pulse duration of 100 μs. The authors confirmed that IRE + CaCl_2_ treatment led to almost double the lesion size compared to IRE + NaCl control. Moreover, the electric field threshold needed to kill the cancer cells has been reduced by ~50%, suggesting that larger lesions may be damaged without the need to increase the applied energy. Novickij et al., 2019 [72] investigated the feasibility of sub-microsecond range IRE + CaCl_2_ in vivo [72]. The authors used two pulsed electric fields (PEF) protocols, PEF1: 12 kV/cm × 200 ns × 500 (0.006 J/pulse) and PEF2: 12 kV/cm × 500 ns × 500 (0.015 J/pulse), both generated at 100 Hz and with or without Ca^2+^ (168 mM). The IRE + CaCl_2_ prolonged the delay in tumor renewal. The applied IRE-based therapy revealed significant effectiveness against primary tumors and induced an antitumor immune response. Unfortunately, it did not enable sufficient control of metastases.

Rudno-Rudzińska et al., 2021 [72] analyzed IRE alone or combined with cisplatin or CaCl_2_ as new treatment strategies for human pancreatic cancer [73]. The pilot preclinical study included 13 patients (7 males and 6 females) with different pancreatic cancer stages. Patients underwent IRE or ECT with intravenous admission of cisplatin (1 mg/mL) or electroporation with intra-tumoral CaCl_2_ (9 mg/mL) administration. The results revealed that the effectiveness of IRE might be enhanced by calcium as an adjuvant. There is an ongoing IREC clinical trial, assuming the recruitment of 70 patients with unresectable pancreatic cancer [74], and a paper reporting 2 cases included in this trial was published [75]. The research findings suggest that calcium (Ca^2+^) administration should be performed after the irreversible electroporation (IRE) procedure, instead of before it, due to the alterations in tissue conductivity brought on by calcium.

In conclusion, combining IRE with chemical substances or cytotoxic agents gives promising results and may enable the ablation of a larger tumor area with lowered energy. It would improve the efficacy and safety of IRE therapy; however, the idea needs to be analyzed more precisely, especially in vivo, using a more extensive sample examination.

**Table 2:** Preclinical and clinical studies involving IRE in combination with chemotherapy.

Type of Trial (Status)	Trial Identifier	Phase	Patients Number	Short Description	Study Protocol	Study Outcomes	Side Effects	Ref.
Randomized (ongoing)	KB-330/2018	N/A	13	IRE + CaCl_2_ or CT in LAPC patients	i.t. CaCl_2_ (10 mM) + IRE: 10 pulses, 1.05–2.8 kV/cm, +/− intraoperative CSP	N/A	N/A	[73,75]
Interventional Clinical Trial	NCT04093141	N/A	30	CT followed by IRE in patients with unresectable LAPC	N/A	N/A	N/A	N/A
Non-randomized Prospective	NCT02981719	N/A	68	LAPC patients treated with GEM with concurrent IRE (*n* = 33) or GEM alone (*n* = 35)	i.v. GEM (1 g/m^2^) +/− IRE: 90 pulses, 1.5 kV/cm, PD = 70–90 ms + i.v. CT (GEM 1 g/m^2^) 2–4 WKs after IRE ablation 6 courses	GEM: OS = 9.3 M, PFS = 8.3 M GEM + IRE: OS = 19.8 M, PFS = 4.7 M	hypoalbuminemia, hemoglobin reduction	[64]
Interventional Clinical Trial	NCT02514421	I	6	Patients diagnosed with stage III pancreatic cancer treated with IRE prior to CT (GEM + nab-paclitaxel)	IRE +/− i.v. CT (GEM 1) g/m^2^; D: 1, 8, and 15 and nab-paclitaxel 0.125 g/m^2^; D: 1, 8, and 15)	N/A	N/A	N/A
Randomized	NCT03673137	II/III	120	LAPC patients treated with simultaneous GEM and IRE	IRE: 80 pulses, 1.5 kV/cm, PD = 90 ms +/− i.v. GEM (1 g/m^2^)	N/A	N/A	N/A
Prospective	NCT03484299	II	20	Patients with advanced pancreatic cancer treated with IRE in combination with either FOLFIRINOX or GEM	IRE: 100–300 pulses, 3 kV/cm, PD = 100 µs + i.v. GEM or FOLFIRINOX	N/A	N/A	[24]

i.v.—intravenous; i.t.—intratumoral; CSP—cisplatin; GEM—gemcitabine; CaCl_2_—calcium chloride; IRE—irreversible electroporation; OS—overall survival rate; WK—week; D—day; M—months; ms—milliseconds; LAPC—locally advanced pancreatic cancer; CT—chemotherapy; PD—pulse duration.

## 6. Immunomodulatory Effects of IRE

Recent studies show that IRE may be important as a method of stimulating and modulating the response of the immune system [76,77]. IRE effects on immunological stimulation are strongly anticipated in pancreatic cancer, which is a lethal disease with a poor prognosis. The immune response to IRE in pancreatic cancer may vary depending on the individual and the specific details of the treatment. In general, IRE can trigger an immune response by causing the release of antigens (substances that stimulate an immune response) from the cancer cells, which can be recognized by the body’s immune system. The immune response may involve the activation of immune cells such as T-cells and the production of cytokines [60,76,78].

The available research shows that irreversible electroporation induces an anticancer immune response, increasing the immunogenicity of cancer and, in combination with immunotherapy, extending the survival time of cancer patients. As a result of the response to cancer and its therapy, pro-inflammatory cytokines and growth factors are released in the body. The available data indicate that elevated concentrations of interleukins [79], i.e., IL1b [80], IL2 [81], IL6 [81,82], IL8, IL10 [60,81,82], tumor necrosis factor—α (TNF-α) [81] are associated with a poor prognosis for patients with pancreatic cancer. So far, no universal panel of inflammatory markers has been developed that would clearly indicate the progression of the disease or its remission. Additionally, most studies concern animal or in vitro studies; clinical data. Tian et al., 2019 [60] indicated the importance of both local and systemic immune responses. In both cased can be observed increased levels of CD8+ T cells and decreased number of Treg and PD-1+ T cells [82]. Interestingly, He et al. 2021 [77] observed increased levels of IL-4, IL-6, TNF, and IFN-γ in the patients treated with IRE + toripalimab (PD-1) than in the group treated only with IRE [81]. In the other study, patients were exposed to IRE only, and post-therapy were observed increased levels of CD4+ T cells, CD8+ T cells, NK cells, IL-2, C3, C4, and IgG. However, a decreased tendency was noted for Treg cells, IL-6, and IL10 [60]. Zhao et al., 2019 [26] demonstrated in cell and animal models that IRE with anti-PD1, an immune checkpoint blockade, stimulates selective tumor infiltration by CD8+ T cells and prolongs survival in animals with the PDAC model [26]. Valuable observations were delivered by Beitel-White et al., 2019 [73] stating that larger overall changes in output current are associated with more significant decreases in T-cells within 24 h post-treatment. Another group also demonstrated a similar effect in a rodent model of pancreatic cancer, where IRE induced a more significant infiltration of macrophages and T-cells than cryoablation within 24 h [83].

Recent studies have shown that IRE can stimulate an immune response by releasing danger-associated molecular patterns (DAMPs) and tumor-associated antigens (TAAs) from dying cancer cells. This process can activate immune cells, such as dendritic cells and macrophages, which can then present the TAAs to T cells, leading to an immune response against cancer [84]. The available studies have demonstrated that IRE can enhance the immune response in various cancers. It was found that IRE increased the levels of CD8+ T cells and cytokines, such as interferon-gamma and tumor necrosis factor-alpha in cancer cells [85,86]. However, it is important to note that these findings are based on preclinical studies in animal models and that more research is needed to determine the safety and efficacy of IRE in pancreatic cancer treatment in humans. Additionally, it is important to consider that the immune response can vary between patients and that other factors, such as the stage and type of cancer, can impact the effectiveness of IRE in potentiating the immune response.

### IRE and CAR-T Therapy

Research was also performed research with the use of CAR-T therapy in combination with IRE for the treatment of pancreatic cancer. Chimeric antigen receptor T-cell (CAR-T) therapy is a type of immunotherapy that involves engineering a patient’s own T-cells (a type of immune cell) to recognize and attack cancer cells. The main challenge for CAR T-cell immunotherapy in pancreatic cancer and other solid tumors is the identification of unique tumor antigens, which may serve as target molecules. To date, several antigens have been investigated as target proteins for CAR-T cells (including mesothelin, CEA, HER2, etc.). Initial trials confirm the effectiveness of the CAR approach in pancreatic cancer treatment with the antigens mentioned above. The first results were reported by Beatty et al., 2018 [82] using mesothelin-targeted CAR-T-cells. After intravenous and intratumoral infusion, CAR-T-cells were detected in the pancreatic tumor biopsy, which confirmed the infiltration of lymphocytes. This fact confirms that CAR-T-cell therapy for pancreatic cancer is warranted, despite the limiting tumor microenvironment [87]. Multiple clinical trials are currently underway with CEA and MUC-1-directed CAR T-cell therapy.

There was one reported study with the use of similar—CAR-T therapy in combination with IRE. IRE was combined with allogeneic γδ-T cells in LAPC patients. An emerged and enhanced antitumor effect and prolonged survival were observed [88]. However, these are very early, and preliminary findings and more research are needed to confirm the potential benefits of this combination therapy in pancreatic cancer. It is important to note that CAR-T therapy is still considered experimental in the treatment of pancreatic cancer and is not widely available.

The immunomodulatory effects of IRE in pancreatic cancer are not fully understood, and more research is needed to determine how IRE may affect the immune system and its role in the treatment’s effectiveness. The immune response triggered by IRE may contribute to the treatment’s effectiveness in killing cancer cells, but this has not been definitively proven.

## 7. Drawbacks Associated with IRE

Although IRE has undoubtedly shown promise as a treatment option for PC patients, several disadvantages that limit its use in both clinical and in vitro models are associated with IRE.

In the clinical model, the difficulty lies in targeting specific areas. IRE requires precise placement of electrodes, which can be challenging in complex geometries, such as tumors located near sensitive organs. The next problem is associated with pain. IRE can cause pain during the procedure, as the electrical pulses can stimulate nerve endings. Problematic can also be limited tissue penetration, as this method is limited by the depth of tissue penetration, making it unsuitable for deep-seated, big, and irregular tumors [75,89,90].

In the in vitro model, we can distinguish the difficulty in controlling cell death. IRE stimulates apoptosis, necrosis, or other cell death types, making it difficult to assess the kind and intracellular mechanisms induced by IRE. Additionally, the experimental assessment can deliver variability in the response [11]. IRE can produce different results in different cell types, making it difficult to predict the therapeutic effect in various cancers. Thus it is essential to optimize IRE parameters.

IRE modality is not commonly used in combination with other anticancer protocols; up to now, it is inquiring about planning and predicting precisely the final effect. In terms of therapeutic effect when used in combination with other treatments, we can only suspect that:

- Inadequate efficacy: IRE may not provide enough therapeutic effect when combined with other treatments, such as chemotherapy or radiation therapy.
- Enhanced toxicity: The combination of IRE with other treatments may increase the toxicity of the treatment, making it less safe for patients.
- Difficulty in determining optimal dosing: The combination of IRE with other treatments can make it difficult to determine the optimal dosing of each treatment, which may impact the therapeutic effect.

These disadvantages highlight the need for further research and development to improve the safety and efficacy of IRE as a cancer treatment.

## 8. Summary

Finding the best therapy for pancreatic cancer can be a complex process and may involve a combination of different treatments. This process is strongly dependent on the proper diagnosis, cancer staging, considering the available treatment options, and then follow-up care. The specific treatment plan will depend on various factors, including the stage of cancer, the patient’s overall health and medical history, and preferences. The IRE method finds its place among the available treatment options, including surgery, chemotherapy, or radiation therapy. As was shown, IRE protocols can be effectively used with cytostatic drugs and calcium ions. The most promising, despite selective tumor destruction, is the potentiation of the immune response, which indicated IRE as a supportive method for the available anticancer protocols.

## Figures and Tables

**Table 1:** Summary of clinical studies of irreversible electroporation (IRE) in pancreatic cancer (PC).

Study Description/Type	Pulse	Number of Patients (Procedure and Type of IRE)	Tumor Size Med. (Range), cm	Main Findings/Outcomes	Adverse Events/Mortality	Median Overall Survival, mo.	Ref.
Study on the LAPC patients treated with IRE. Ninety-day follow-up for morbidity, mortality, and local disease control/prospective.	M (23) B (4)	27 (19 IRE, 8 IRE + resection; 26-OP, 1P)	3 (1–5.5)	IRE is safe and feasible on PC. No evidence of residual tumor during follow-up. No reports of clinical pancreatitis or fistula formation during the 90-day follow-up/palliation of pain symptoms and reduction of overall narcotic use.	Major 33%/ Mortality 4%	NR	Martin et al., 2012 [39]
IRE performed in PC patients with unresectable tumors. The evaluation of tumor response, adverse events, and survival.	M B	14 (3 with metastatic disease and 11 with LAPC; P)	3.3 (2.5–7)	The authors conclude that IRE is feasible and safe. No survival benefits for patients with metastatic disease/two patients had R0 resection after IRE and stayed disease free 11 and 14 months. No serious complications reported.	NR/NR Patients with the metastatic disease died due to the progression	NR	Narayanan et al., 2012 [40]
Evaluation of overall survival of patients treated with IRE vs. standard therapy (chemotherapy and radiation therapy alone)/prospective.	M (48) B (6)	54 (OP-52, P-2)	3.2 (1–5.5)	Better progression-free survival with greater local palliation. 20.2 mo. of OS with IRE+ chemotherapy vs. 11 mo. after chemotherapy alone.	67	20.2	Martin et al., 2013 [41]
Assessment of the efficacy of IRE as part of multimodal treatment in stage III LAPC patients. In a prospective multicenter study, the determination of perioperative 90-day outcomes, local failure, and OS.	M	200 (150 IRE, 50 IRE+ resection); OP	3 (1.6–7)	IRE on LAPC patients after chemotherapy and chemoradiotherapy resulted in prolonged survival. During 29 months, follow-up six patients have a local recurrence.	37%/NR	24.9	Martin et al., 2015 [23]
Single institution clinical trial, determination of perioperative morbidity and mortality for LAPC patients treated with IRE.	M	50 (29 IRE, 24 margin extension IRE); OP	3.0 (1.7–5)	The mortality and morbidity rates were higher than reported before. There is a need for further research on the safety and efficacy of IRE in PC. IRE should not be considered a minimally invasive treatment.	16/mortality 6 (11%) in 90-day follow-up (5 with IRE)	IRE 7.71	Kluger et al., 2015 [42]
Patients with LAPC with no evidence of metastasis pretreated with chemo-/and or radiotherapy were treated with percutaneous CT-guided IRE.	M	24, P-US-guided	3.5 (1.5–4.5)	Downstaging in two patients, local control in 9 patients. The study confirmed the safety and efficacy of IRE procedure, but more study is needed to improve the outcomes of the procedure.	11 (3 severe, 8 minor)/NR	17.9 (7 after IRE)	Mansson et al., 2016 [43]
Retrospective study to define safety and efficacy of percutaneous IRE for treatment of LAPC.	M (45) B (5)	50, P	3.4 (1–6)	Downstaging in case of 3 patients. Studies indicated that tumor size < 3 is the only objective predictor of overall survival.	45 (35 mild/10 serious)/NR	27 (14.2 from IRE)	Naraynan et al., 2017 [44]
The phase I/II PANFIRE study of ablation with percutaneous IRE of LAPC. Evaluation of safety, efficacy, quality of life, pain perception, event-free survival OS/prospective.	M	25, P	4 (3.3–5)	IRE is generally well tolerated. Only three patients had signs of pancreatitis, although major avert effects can occur. Median event-free survival after IRE—8 mo. Time of local progression 12 mo.	23 (11 major/11minor)/NR	11	Scheffer et al., 2017 [45]
Treatment of patients with unresectable LAPC with CT-guided IRE. The evaluation of postoperative immediate and 30-day morbidity and mortality, progression-free (PFS), and overall survival (OS).	M	75 P	3.47 ± 1.2	Studies showed the safety and ability to combine IRE procedures in unresectable LAPC patients with standard chemotherapy for better OS and PFS. Median progression-free survival post-IRE was 15 mo. Four patients downstage after IRE ablations which allowed R0 resection in 3 cases.	25%/NR	27	Leen et al., 2018 [46]
Multicenter study for evaluation of safety and efficacy of IRE in LAPC patients treated with laparotomic and laparoscopic IRE with prior treatment with gemcitabine or TS-1 chemotherapy.	NA	70 (65 OP, 5 laparoscopic)	NA	IRE is safe and effective for the control of LAPC. The addition of IRE to a chemotherapy regimen improves survival. PFS 15.4 mo. (13.2 in the gemcitabine group, 26.4 in the TS-1 group).	30 (3 major, 27 minor)/NR	22.6 (19.1 gemcitabine-based reagents, 28.7 TS-1)	Sun et al., 2021 [25]
Determination of safety and efficacy of IRE with or without chemotherapy for unresectable pancreatic carcinoma (stage III/IV). Prospective	M	54; OP, P	4.9	Pancreatic cancer patients may benefit from IRE, which has improved OS in certain patients who also received chemotherapy. IRE was generally well tolerated. IRE can provide local tumor control with relatively satisfactory PFS and OS in stage III and IV pancreatic cancer patients with large tumors (>5 cm).	4 major/44 minor	16.6 20.3 IRE + chemotherapy	Liu et al., 2019 [47]
A multicenter, prospective phase II study of percutaneous IRE for LAPC and recurrent PC.		50 (40 LAPC, 10 local recurrences); CT guided IRE, P	3.7	IRE should be considered a high-risk procedure due to the possibility of major adverse effects. The study suggests survival benefits from IRE compared to standard-of-care.	21 major, 14 minor/2 deaths within 90 days of IRE	17 from diagnosis 11.6 IRE 14.9 IRE+ FOLFIRINOX	Ruarus et al., 2020 [48]

Abbreviations: IRE; irreversible electroporation, M; monopolar, B; bipolar, OP; open, P; percutaneous, OS; overall survival rate, mo; months, LAPC; locally advanced pancreatic cancer; PFS, progression-free survival, NA; not available, NR; not reported.

## Data Availability

Not applicable.
